# Apport de la simulation dans l’apprentissage de la cardiologie interventionnelle chez les débutants: étude tunisienne transversale

**DOI:** 10.11604/pamj.2023.46.119.36874

**Published:** 2023-12-28

**Authors:** Rania Hammami, Jihen Jdidi, Amine Bahloul, Tarek Ellouze, Sahar Kmiha, Oussema Haddar, Yacine Abdelmoula, Majed Hassine, Aymen Dammak, Selma Charfeddine, Mootaz Billah Oueslati, Mahdi Ben Dhaou, Leila Abid

**Affiliations:** 1 Service de Cardiologie, Centre Hospitalier Universitaire Hedi Chaker, Faculté de Médecine de Sfax, Université de Sfax, Sfax, Tunisie,; 2 Centre de Simulation de la Faculté de Médecine de Sfax, Université de Sfax, Sfax, Tunisie,; 3 Service d´Epidémiologie, Centre Hospitalier Universitaire Hedi Chaker, Faculté de Médecine de Sfax, Université de Sfax, Sfax, Tunisie,; 4 Service de Cardiologie A, Centre Hospitalier Universitaire Fattouma Bourguiba de Monastir, Cardiothrombosis Research Laboratory (LR12SP16), Université de Monastir, Monastir, Tunisie,; 5 Service de Chirurgie Cardiovasculaire, Centre Hospitalier Universitaire Habib Bourguiba Sfax, Université de Sfax, Sfax, Tunisie,; 6 Service de Chirurgie Pédiatrique, Centre Hospitalier Universitaire Hedi Chaker Sfax, Université de Sfax, Sfax, Tunisie

**Keywords:** Simulation, cardiologie interventionnelle, performance, compétence, apprenants, Simulation, interventional cardiology, performance, competence, learners

## Abstract

**Introduction:**

l´apprentissage de la cardiologie interventionnelle basée sur la simulation (ABS) est un outil éducatif en plein essor dans le monde entier. Nous avons mené alors cette étude afin d´évaluer l´impact de l´ABS sur l´évolution des compétences des apprenants débutants dans le domaine, à travers un cycle de formation de faible durée.

**Méthodes:**

nous avons mené une étude évaluative quasi-expérimentale de type avant-après dans le centre de simulation de la Faculté de Médecine de Sfax. Nous avons impliqué des résidents en cardiologie au début de leur formation en cardiologie interventionnelle. Tous les participants ont bénéficié d´un cycle de formation ayant duré 4 heures sur un simulateur à haute-fidélité de type Cathi^®^, après avoir donné leurs consentements. Le critère de jugement primaire a été définie par l´amélioration significative des scores de performance et de compétence avant et après le cycle. Le critère de jugement secondaire a été défini par la réduction de temps d´irradiation et le temps de la procédure.

**Résultats:**

treize apprenants ont participé dans notre étude. Le score de performance s´est amélioré d´une médiane de 216,12% (ISQ= 285%). Cette amélioration était significativement plus nette chez les apprenants qui n´ont jamais eu accès à la salle de cathétérisme. Le score de performance a évolué d´une médiane de 31 (ISQ=40,5) à une médiane de 120 (ISQ=19,7), (p=0,001). Le score de compétence lié à la coronarographie s´est amélioré significativement, d´une médiane de 16 (ISQ=18) vers une médiane de 70 (ISQ=6), (p=0,001). Le score de compétence lié à l´angioplastie a évolué significativement d´une médiane de 10 (ISQ=17) vers une médiane de 50 (ISQ=13,7), p=0,001. Les durées de la coronarographie et de l´angioplastie ont été nettement réduites ayant évolué respectivement de 12 min (ISQ=2) à 7 min (ISQ=1) après le cycle de simulation (p=0,001), et d´une médiane de 19 min à une médiane de 17 min après la simulation, p= 0,002.

**Conclusion:**

malgré la durée courte de la formation par simulation, notre étude Pilote a démontré une amélioration significative des compétences et performances des apprenants ainsi qu’un gain de temps de la réalisation des procédures et d´irradiation ce qui pourrait augmenter éventuellement le nombre de procédures réalisées quotidiennement dans notre cathlab et limiter l´irradiation du personnel et des patients tout en assurant une formation adéquate aux apprenants.

## Abstract (en)

**Introduction:**

simulator training in interventional cardiology (ST) is an educational tool that is rapidly spreading worldwide. The purpose of this study was to evaluate the advantages of ST in improving skills of beginners in interventional cardiology procedures, through a short training cycle.

**Methods:**

we conducted a before-and-after quasi-experimental evaluative study in the simulation center of the Faculty of Medicine in Sfax. We involved cardiology residents at the beginning of their training in interventional cardiology. All the participants attended a 4-hour training course on a Cathi^®^, high-fidelity simulator, after giving their consent. The primary endpoint was a significant improvement in performance and competence scores before and after the course. The secondary endpoint was the reduction in irradiation time and the time of the procedure.

**Results:**

thirteen learners participated in our study. The performance score improved by a median of 216.12% (ISQ = 285%). This improvement was significantly greater for learners who had never had access to the catheterisation room. The performance score ranged from a median of 31 (ISQ=40.5) to a median of 120 (ISQ=19.7), (p=0.001). The competence score for coronary angiography improved significantly, from a median of 16 (ISQ=18) to a median of 70 (ISQ=6), (p=0.001). The competence score for angioplasty improved significantly from a median of 10 (ISQ=17) to a median of 50 (ISQ=13.7), p=0.001. Procedure time of coronary angiography and angioplasty were significantly shortened from 12 min (ISQ=2) to 7 min (ISQ=1) after the simulation cycle (p=0.001), and from a median of 19 min to a median of 17 min after simulation, p=0.002.

**Conclusion:**

despite a short-time simulation training, our pilot study demonstrates a significant improvement in the learners´ skills and performance, as well as a reduction in the time taken to carry out procedures and irradiation. This could eventually increase the number of procedures carried out daily in our cathlab and limit radiation exposure of staff and patients, while ensuring that the learners receive adequate training.

## Introduction

La simulation en santé est devenue incontournable dans l´apprentissage des différents domaines de cardiologie en particulier la Cardiologie Interventionnelle (CI). Cette pratique est organisée de nos jours par des recommandations internationales [1-4]. Une enquête récente internationale a montré que seulement 24% des salles de cathétérisme disposent d´un plateau de simulation et la durée allouée dans ces centres aux cycles de formation par simulation varie entre 2 à 10 jours par an [3]. Dans certains pays comme les Etats-Unis, la simulation est devenue obligatoire pour l´accréditation des internes ainsi que pour le renouvellement de licences de pratiques de la CI [1,3,5,6]. La survenue de la pandémie de COVID-19 a été un nouveau motif qui a incité les décideurs dans le domaine de la santé, dans plusieurs pays à développer l´apprentissage basé sur la simulation (ABS). En effet, l´activité des salles de cathétérisme durant cette pandémie a été réduite de 50 à 75%, ce qui a entravé le cursus de formation de plusieurs « apprenants » à cette époque [7-11]. La plupart des sociétés savantes ont recommandé en effet de réduire le nombre d´intervenants dans les salles de cathétérisme cardiaque afin de limiter le risque de contamination [12,13]. La mise en place d´un plateau technique de simulation aurait permis de diminuer le préjudice de cette pandémie sur la formation en CI, de nos apprenants [2,14,15]. Nous avons alors mené cette étude pilote afin d´évaluer l´impact d´un cycle de formation de courte durée moyennant un simulateur à haute-fidélité dans l´évolution des compétences et performances des apprenants dans le domaine de la CI.

## Méthodes

**Design de l´étude**: il s´agit d´une étude transversale évaluative quasi-expérimentale de type avant -après, réalisée au niveau du centre de simulation de la Faculté de médecine de Sfax, au mois de mai 2022. Nous avons évalué l´efficacité d´une séance de formation utilisant un simulateur de CI dans l´apprentissage de procédures invasives (coronarographie et angioplastie), chez les débutants dans la spécialité.

**Figure 1:**
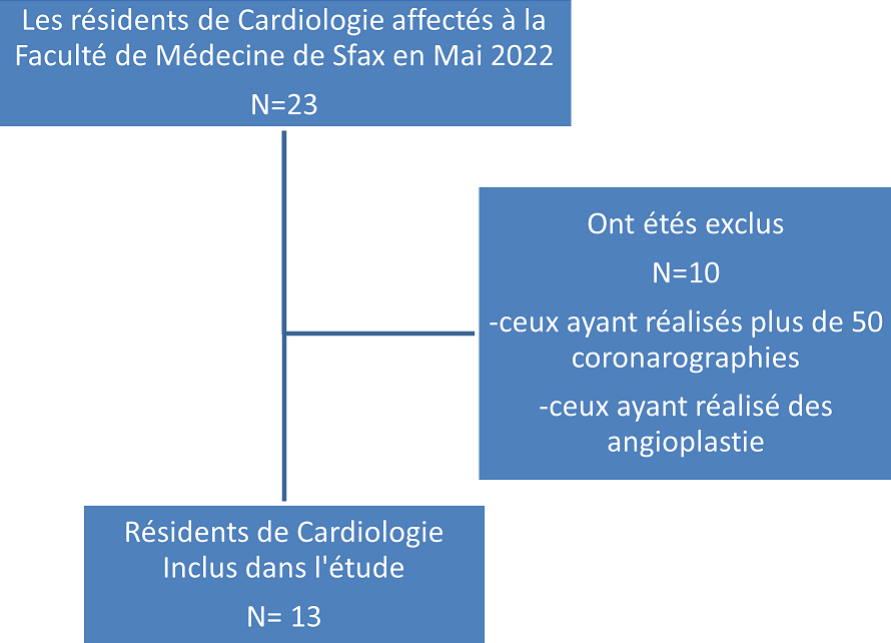
diagramme flow de l´étude

**Participants**: les participants étaient des résidents de cardiologie, au début de leurs formation de CI. Ceux qui ont déjà réalisé plus de 50 coronarographies en tant que premier opérateur ou ceux ayant réalisé des angioplasties ont été exclus de l´étude (Figure 1). Tous les participants ont donné leur consentement écrit pour participer dans cette formation après avoir expliqué le scenario de la formation. Le protocole de l´étude a été approuvé par le comité d´éthique de la Faculté de Médecine de Sfax, sous le numéro 16/23.

**Figure 2:**
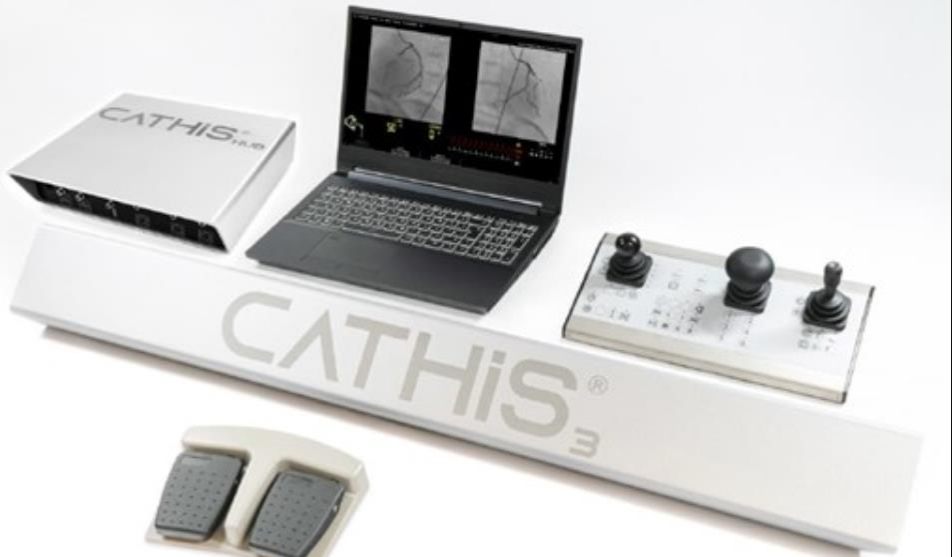
simulateur de type “SMART” de CATHI

**Conception de l´étude**: nous avons utilisé un simulateur de marque CATHI de type « SMART » (Figure 2), loué par la Faculté de Médecine de Sfax pour l´année universitaire 2021/2022. Le simulateur est formé des composantes suivantes : une unité de contrôle permettant de modifier l´incidence de projection, de contrôler les mouvements de la table, de changer l´agrandissement, un ordinateur « Note Book » ou s´affiche la procédure, la durée de la fluoroscopie, les paramètres hémodynamiques du patient, une pédale de scopie, et un instructeur d´instruments qui permet de choisir les instruments (ballon, stent)

**Scenario de simulation**: le premier jour nous avons collecté les données concernant l´expérience de tous les participants en fonction du nombre de procédures réalisées pour chaque participant (classées en 3 groupes: 0 coronarographie, entre 0 et 25 coronarographies, entre 26 et 50 coronarographies), la durée de stage réalisé dans une salle de cathétérisme. Un opérateur ayant déjà manipulé le simulateur avait expliqué les différentes composantes du simulateur et a réalisé une procédure de coronarographie et d´angioplastie coronaire en présence de tous les apprenants. Le cycle de formation s´est déroulé de la façon suivante: chaque participant a bénéficié d´une séance de simulation ayant duré quatre heures de temps pour manipuler le simulateur. Tous les apprenants ont été supervisés par le même cardiologue. Pendant la durée de la formation l´apprenant pouvait réaliser 5 cas de coronarographies et 5 cas d´angioplasties, selon la rapidité de son apprentissage.

**Evaluation des apprenants**: les apprenants ont été évalués en deux temps en calculant un score « de performance ». Ce score est composé de 30 items: 14 items pour évaluer le déroulement de la coronarographie (score de « compétence » en coronarographie), 14 items pour évaluer le déroulement de l´angioplastie (score de « compétence » en angioplastie) et 2 items d´habiletés (Annex 1). Ces scores sont calculés au début de la formation lors de la réalisation de la première coronarographie et la première angioplastie. Le lendemain de la formation l´apprenant revient pour passer le post-test qui consiste à réaliser la même coronarographie et la même angioplastie avec laquelle il a commencé. Nous avons défini un succès procédural pour la coronarographie par la réalisation de la coronarographie du post-test en moins de 10 min ; et nous avons défini un succès procédural pour l´angioplastie par la réalisation de l´angioplastie du post-test en moins de 30 min.

**Le critère de jugement principal**: nous avons comparé les scores de performances et de compétences avant et après le cycle de simulation. Nous avons calculé le pourcentage de la variation du score de performance chez tous les participants (100*(Score final-score initial)/score initial).

**Les critères secondaires de jugement**: nous avons considéré comme critères secondaires de jugement la variation de la durée fluoroscopie de la coronarographie et de l´angioplastie, ainsi que la variation des durées déroulement des procédures de coronarographies et d´angioplasties.

**Analyse statistique**: nous avons utilisé le logiciel SPSS, IBM® version 21, pour établir les statistiques. Etant donné le faible effectif de l´échantillon, nous avons considéré d´emblée que la distribution de notre population est non gaussienne et nous avons exprimé toutes les valeurs quantitatives en médiane (avec les extrêmes et l´intervalle semi interquartile (ISQ)) et nous avons utilisé les tests non paramétriques (le test de Wilcoxon) afin de comparer les scores de performance et de compétence avant et après cycle de formation (tests d´échantillons appariés). La comparaison des scores entre ceux qui ont n´ont jamais fait de coronarographies (les novices) et ceux qui ont déjà pratiqué des coronarographies a été faite par le test non paramétriques de Mann Witney. Nous avons considéré que la différence est significative si p< 0,05.

## Résultats

**Caractéristiques générales de la population**: treize résidents de cardiologie ont participé dans cette étude dont la majorité était des résidents en première année de cursus de formation. La majorité était de sexe masculin, et n´avaient par ailleurs aucune expérience technique dans la réalisation de la coronarographie (Tableau 1).

**Tableau 1:** expérience des participants dans notre étude

**Nombre de participants**	13
**Sexe masculin**	9/13
**Année du cursus**	1^ère^ année: 9/13
	2^è^ année: 3/13
	3^è^ année: 1/13
**Réalisation de stage dans une salle de cathétérisme**	4/13
**Apprenants ayant assisté à au moins 5 procédures (angioplastie primaire)**	6/7
**Nombre de coronarographies réalisées auparavant**	Aucune coronarographie: 9/13
	Moins de 25 coronarographies: 2/13
	Entre 26 et 50 coronarographies: 2/13
**Nombre de coronarographies réalisées lors de la formation**	3 coronarographies: 3/13
	4 coronarographies: 4/13
	5 coronarographies: 6/13
**Nombre d´angioplasties réalisées lors de la formation**	2 angioplasties: 5/13
	3 angioplasties: 8/13

**Critères primaires de jugement**: concernant le succès procédural, nous avons noté qu´avant le cycle de simulation, 4/13 participants n´ont pas pu réaliser la coronarographie dans les délais requis (<10 min), de même 3/13 n´ont pas pu réaliser l´angioplastie dans les délais requis (<30 min). Après le cycle de simulation, tous les participants ont pu réaliser la coronarographie et l´angioplastie dans les délais requis. Dans toute la population, le score de performance s´est amélioré d´une médiane de pourcentage de 216,12% (ISQ= 285%). Les scores de compétences de la coronarographie, de l´angioplastie ainsi que le score de performance se sont améliorés de façon significative après le cycle de formation (Tableau 2). Le pourcentage de l´amélioration de score de performance était significativement plus élevé chez les participants qui n´ont jamais réalisé de coronarographie (les novices) comparée à ceux qui ont déjà réalisé une coronarographie (361,5% (ISQ=260) versus 36,5% (ISQ=12,5), p=0,02 (Tableau 3).

**Tableau 2:** évolution des scores de compétences obtenus par les apprenants après la formation de simulation

		Population totale			Chez les novices (N=9)		
		Avant	Après	P	Avant	Après	P
**Compétence de la Coronarographie (Par rapport 70)**	**Médiane**	16	70	0,001	11	65	0,008
	**ISQ**	18	6		14	9,2	
	**Extrêmes**	(5- 55)	(43- 70)		(5-33)	(43-70)	
**Compétence de l’Angioplastie (Par rapport à 70)**	**Médiane**	10	50	0,001	10	40	0,008
	**ISQ**	17	13,7		5,7	10	
	**Extrêmes**	(0- 55)	(25- 65)		(0-25)	(25-65)	
**Score d'habileté (Par rapport à 10)**	**Médiane**	5	10	0,004	5	10	0,006
	**ISQ**	2,7	-		2	-	
	**Extrêmes**	(0- 10)	(10)c		(0- 5)	(10)c	
**Score de performance final (Par rapport à 150)**	**Médiane**	31	120	0,001	26	118	0,008
	**ISQ**	41,2	19,7		12,5	14.2	
	**Extrêmes**	(7-110)	(78-145)		(7-63)	(78-145)	

**Tableau 3:** pourcentage d´amélioration des scores de la performance totale et de la compétence en coronarographie, angioplastie et habileté (%)

		Total (N=13)	Les novices (N=9)	Les autres (N=4)	P
**Amélioration du score de la Compétence en Coronarographie, (%)**	**Médiane**	233	427	47,77	0,003
	**ISQ**	242	260	12,5	
**Amélioration du score de la Compétence en Angioplastie, (%)**	**Médiane**	200	300	33,7	0,003
	**ISQ**	845	1200	20,9	
**Amélioration du score de l´habileté, (%)**	**Médiane**	100	100	0	0,02
	**ISQ**	220	400	37,5	
**Score de performance final, (%)**	**Médiane**	216,12	361,5	36,5	0,003
	**ISQ**	285	260	12,5	

**Figure 3:**
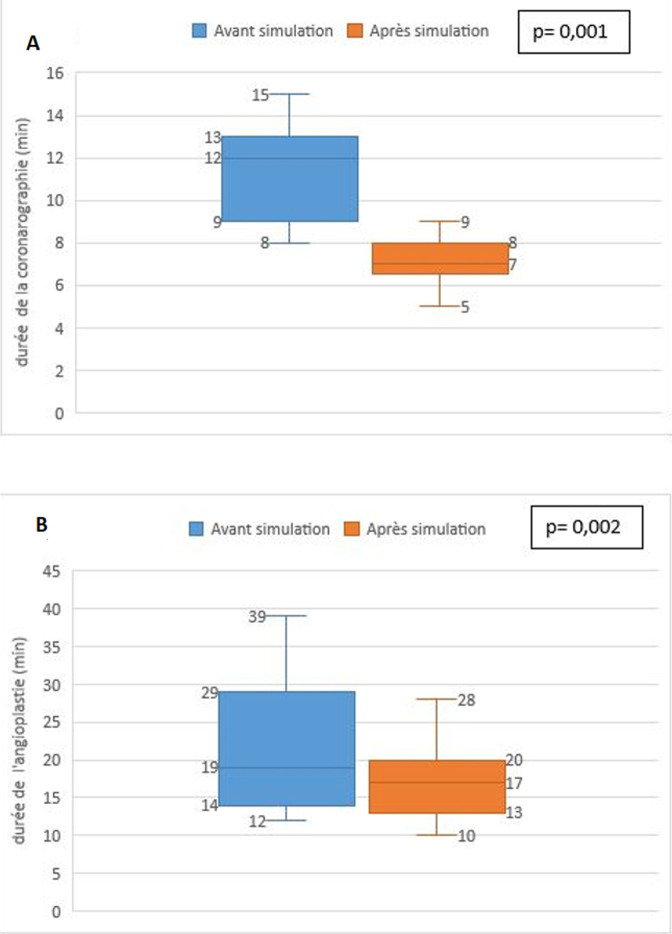
évolution des durées procédurales avant et après le début du cycle de formation (A) durée de la coronarographie, (B) durée de l´angioplastie

**Critères secondaires de jugement**: les durées procédurales ont nettement régressé passant d´une médiane de 12 min (ISQ=2) avant le cycle de simulation à une médiane de 7 min (ISQ=1) après la simulation (p= 0,001) pour la coronarographie et d´une médiane de 19 min avant le cycle de simulation à une médiane de 17 min après la simulation pour l´angioplastie (p= 0,002) (Figure 3). La durée de la fluoroscopie après l´angioplastie a nettement régressé passant d´une médiane de 568 (ISQ=150,5) secondes avant le cycle de simulation à une médiane de 520 (ISQ=120) secondes après la simulation, p= 0,034. Par contre, la durée de la fluoroscopie après coronarographie n´a pas été réduite après le cycle de simulation. Elle était de 312 (ISQ=43) secondes avant le cycle et 312 (ISQ=52) secondes après le cycle de formation (p=0,15).

## Discussion

La présente étude est un essai pilote de l´évaluation de l´Acrylonitrile-butadiene-styrene (ABS) dans le domaine de la Cardiologie Interventionnelle en Tunisie, dont l´objectif est d´évaluer l´impact d´un cycle de formation par simulation de courte durée sur l´amélioration des performances et compétences des apprenants. Nous avons démontré que cette pratique a amélioré le score de performance d´une médiane de 216%. Cette amélioration était significativement plus importante chez les résidents qui n´ont jamais réalisé de coronarographies comparées à ceux qui ont déjà réalisé quelques procédures de coronarographies. Les séries qui ont évalué l´ABS dans l´apprentissage de la CI sont de nombre limité et ont inclus de faibles effectifs. Tous les essais étaient de type prospectif et ont adopté deux méthodologies différentes : certains essais ont évalué l´amélioration des performances des apprenants avant et après un cycle d´ABS utilisant ainsi le modèle d´échantillons appariés tel était le cas de notre étude, d´autres essais étaient de type expérimental et ont comparé un groupe d´apprenants ayant bénéficié de l´ABS à un groupe témoin. Le faible effectif était la limite majeure de toutes les séries [16-20]. Les critères d´évaluations adoptés dans les différentes séries étaient hétérogènes du fait du manque de recommandations concernant l´évaluation de cette pratique. Voelker *et al*. ont choisi de filmer les manipulations du matériel par les apprenants et ont évalué par la suite leurs compétences en se basant sur 14 items. Le score de performance a été calculé avant et après le cycle de formation par simulation, tout en comparant deux groupes: un groupe de simulation et un groupe de formation théorique. Le score a été significativement amélioré dans le groupe « simulation » par contre il a été significativement réduit dans le groupe témoin [19]. Schimmel *et al*. ont utilisé un score de performance composé de 32 items pour la voie radiale et 33 items pour la voie fémorale [21]. Certains items évaluaient les connaissances des participants (en particulier la radioprotection), d´autres évaluaient les aspects techniques des procédures. Bagai *et al*. ont comparé deux groupes, un groupe « simulation » ayant reçu un enseignement théorique et une formation de 4 heures de simulation, et un groupe « témoin » d´apprenants ayant bénéficié seulement d´un enseignement théorique [22]. L´évaluation était basée sur un score évaluant en particulier l´habilité technique mais aussi les connaissances concernant les incidences qui permettent de dégager les différents segments coronaires. Malgré l´hétérogénéité des moyens d´évaluations et des cycles de formations, toutes les études ont conclu à une amélioration nette des compétences et des connaissances des candidats après l´ABS [23].

Dans notre série nous avons adopté un score de 30 items évaluant à la fois l´étape de réalisation d´une coronarographie et d´une angioplastie mais aussi l´habilité du participant en évaluant le succès des procédures en un temps bien précis. La version du simulateur Cathi utilisé dans notre étude ne nous renseigne pas malheureusement sur la quantité de produit de contraste utilisé, d´autres versions plus sophistiquées offrait cette option. Nous avons réussi à démontrer une amélioration du score de compétence ainsi que celui de la performance, malgré la courte durée de la formation par simulation, limitée à 4 Heures. Cette amélioration était plus significative chez les touts débutants, n´ayant jamais touché à une procédure de coronarographie, ce qui témoigne de l´importance de l´initiation des formations prodiguées sur simulateur. En outre, nous avons démontré une réduction significative de la durée des procédures ainsi que celle de la fluoroscopie au cours des procédures d´angioplasties. La notion de radioprotection demeure importante en matière de CI [23]. Sur une carrière de 30 ans, un cardiologue interventionnel recevra 900 mSv ce qui majorera le risque de néoplasie de 3,6% par an [24]. L´un des préjudices de l´apprentissage conventionnel en salle de cathétérisme est ainsi l´irradiation importante des opérateurs. Développer la simulation en tant que moyen d´apprentissage est bénéfique non seulement pour l´apprenant mais aussi pour l´opérateur superviseur, le personnel de la salle de cathétérisme et le patient [25]. L´une des limites de la plupart des études évaluatives de l´ABS réside dans l´absence d´évaluation de l´impact clinique de cette pratique (taux de complications, durée de scopie et irradiation, durée des procédures ultérieurement dans un cathlab). Prenner *et al*. étaient avant-gardiste dans ce domaine et ont analysé l´impact de l´ABS dans la « vraie vie » en analysant 2783 coronarographies réalisées par 32 apprenants selon la formation reçue avant de commencer leurs stages en salle de cathétérisme: 12 apprenants avaient bénéficié d´un ABS sur un simulateur VIST-C de MENTICE et 20 apprenants étaient formés de façon conventionnelle. Les procédures effectuées par ceux formés par un ABS étaient plus courtes (moyenne de 23,98 min vs 24,94 min, P = 0,034) avec une diminution significative du rayonnement (moyenne de 56 348 mGycm^2^ vs 66 120 mGycm^2^, p< 0,001). Après élimination des facteurs de confusions, l´apprentissage sur simulateur était associée indépendamment à une réduction significative de 117 secondes du temps de fluoroscopie par procédure (p= 0,04) [16].

Les limites de notre étude consistent aux faibles effectifs des participants, le manque de groupe témoin et l´absence d´évaluation de l´impact de ce cycle dans la vraie vie au cathlab. La simulation en santé étant une méthode d´apprentissage active basée sur la supervision clinique, elle nécessite par conséquent la mobilisation d´importantes ressources humaines avec la disponibilité d´un plateau technique adéquat. Il s´agit d´une méthode chronophage qui nécessite une motivation à la fois de l´apprenant et du superviseur, ce qui explique le faible effectif de participants dans la plupart des études menées.

## Conclusion

Notre étude a confirmé le bénéfice pédagogique de la formation par simulation même de courte durée sur l´évolution des compétences et performances des apprenants en Cardiologie Interventionnelle. En outre, nous avons démontré un gain du temps de la réalisation de ces procédures ainsi que celui l´irradiation, ce qui pourrait être retentir dans le futur sur l´activité quotidienne de notre cathlab recevant plus de 10 apprenants par an et limiter de l´irradiation et du patient et de tout le personnel.

## Annexe

**Annex 1**: les items du score de performance utilisé

## References

[1] Beller GA, Bonow RO, Fuster V, Core Cardiology Training Symposium (COCATS) (2002). ACC revised recommendations for training in adult cardiovascular medicine. Core Cardiology Training II (COCATS 2) (Revision of the 1995 COCATS training statement). J Am Coll Cardiol.

[2] Chong JH, Chahal CAA, Gupta A, Ricci F, Westwood M, Pugliese F (2021). Covid-19 and the digitalisation of cardiovascular training and education: a review of guiding themes for equitable and effective post-graduate telelearning. Front Cardiovasc Med.

[3] Green SM, Klein AJ, Pancholy S, Rao S V, Steinberg D, Lipner R (2014). The current state of medical simulation in interventional cardiology: a clinical document from the Society for Cardiovascular Angiography and Intervention´s (SCAI) Simulation Committee. Catheter Cardiovasc Interv.

[4] Hunter S, Tynan S, Silove E (1996). Guidelines for specialist training in paediatric cardiology. Heart.

[5] Westerdahl DE, Henry TD (2016). "More may mean less." the role for simulation-based medical education in the cardiac catheterization laboratory. Catheter Cardiovasc Interv.

[6] Li S, Qin J, Guo J, Chui Y-P, Heng P-A (2011). A Novel FEM-Based Numerical Solver for Interactive Catheter Simulation in Virtual Catheterization. Int J Biomed Imaging.

[7] Garcia S, Albaghdadi MS, Meraj PM, Schmidt C, Garberich R, Jaffer FA (2020). Reduction in st-segment elevation cardiac catheterization laboratory activations in the United States during COVID-19 pandemic. J Am Coll Cardiol.

[8] Metzler B, Siostrzonek P, Binder RK, Bauer A, Reinstadler SJ (2020). Decline of acute coronary syndrome admissions in Austria since the outbreak of COVID-19: the pandemic response causes cardiac collateral damage. Eur Heart J.

[9] Silva PGM de B E, Dutra AAF, Manfredi AB, Sampaio PPN, Correa CM, Griz HB (2021). Reduction in the number of patients with suspected and confirmed acute coronary syndrome during the early months of the covid-19 pandemic: analysis of a brazilian network. Arq Bras Cardiol.

[10] Boukhris M, Azzalini L, Baystrukov V, Aloui H, Kretov E, Ribeiro MH (2021). Impact of the COVID-19 pandemic on acute coronary syndrome and stroke volumes in non-western countries. Cardiovasc Revascularization Med Mol Interv.

[11] Ibdah R, Rawashdeh S, Al-Kasasbeh A, Albalas M, Obeidat NA, Alrabadi N (2012-2020). The impact of COVID-19 pandemic on the cardiology services in Northern Jordan. Ann Med Surg.

[12] Mahmud E, Dauerman HL, Welt FGP, Messenger JC, Rao S V, Grines C (2020). Management of acute myocardial infarction during the covid-19 pandemic: a position statement from the society for cardiovascular angiography and interventions (SCAI), the American College of Cardiology (ACC), and the American College of Emergency Physicians (ACEP). J Am Coll Cardiol.

[13] Mahmud E, Dauerman HL, Welt FGP, Messenger JC, Rao S V, Grines C (2020). Management of acute myocardial infarction during the COVID-19 pandemic: a consensus statement from the society for cardiovascular angiography and interventions (SCAI), the American College of Cardiology (ACC), and the American College of Emergency Physicians (ACEP). Catheter Cardiovasc Interv.

[14] Yadav A (2020). Cardiology training in times of COVID-19: beyond the present. Indian Heart J.

[15] Chong JH, Chahal A, Ricci F, Klarich K, Ferrari V, Narula J (2021). The transformation of cardiology training in response to the COVID-19 pandemic: enhancing current and future standards to deliver optimal patient care. Can J Cardiol.

[16] Prenner SB, Wayne DB, Sweis RN, Cohen ER, Feinglass JM, Schimmel DR (2018). Simulation-based education leads to decreased use of fluoroscopy in diagnostic coronary angiography. Catheter Cardiovasc Interv.

[17] Jensen UJ, Jensen J, Olivecrona GK, Ahlberg G, Tornvall P (2013). Technical skills assessment in a coronary angiography simulator for construct validation. Simul Healthc.

[18] Jensen UJ, Jensen J, Ahlberg G, Tornvall P (2016). Virtual reality training in coronary angiography and its transfer effect to real-life catheterisation lab. EuroIntervention.

[19] Voelker W, Petri N, Tönissen C, Störk S, Birkemeyer R, Kaiser E (2016). Does simulation-based training improve procedural skills of beginners in interventional cardiology? a stratified randomized study. J Intervent Cardiol.

[20] Casey DB, Stewart D, Vidovich MI (2016). Diagnostic coronary angiography: initial results of a simulation program. Cardiovasc Revasc Med.

[21] Schimmel DR, Sweis R, Cohen ER, Davidson C, Wayne DB (2016). Targeting clinical outcomes: endovascular simulation improves diagnostic coronary angiography skills (Targeting Clinical Outcomes). Catheter Cardiovasc Interv.

[22] Bagai A, O´Brien S, Al Lawati H, Goyal P, Ball W, Grantcharov T (2012). Mentored simulation training improves procedural skills in cardiac catheterization: a randomized, controlled pilot study. Circ Cardiovasc Interv.

[23] Jensen UJ, Jensen J, Olivecrona G, Ahlberg G, Lagerquist B, Tornvall P (2014). The role of a simulator-based course in coronary angiography on performance in real life cath lab. BMC Med Educ.

[24] Gutierrez-Barrios A, Cañadas-Pruaño D, Noval-Morillas I, Gheorghe L, Zayas-Rueda R, Calle-Perez G (2022). Radiation protection for the interventional cardiologist: practical approach and innovations. World J Cardiol.

[25] Schimmel DR, Sweis R, Cohen ER, Davidson C, Wayne DB (2016). Targeting clinical outcomes: endovascular simulation improves diagnostic coronary angiography skills. Catheter Cardiovasc Interv.

